# Multifunctional exosomes derived from bone marrow stem cells for fulfilled osseointegration

**DOI:** 10.3389/fchem.2022.984131

**Published:** 2022-08-22

**Authors:** Jingwen Zhuang, Ruiyue Hang, Ruoyue Sun, Yanshu Ding, Xiaohong Yao, Ruiqiang Hang, Hui Sun, Long Bai

**Affiliations:** ^1^ Key Laboratory for Ultrafine Materials of Ministry of Education, East China University of Science and Technology, Shanghai, China; ^2^ Shanxi Key Laboratory of Biomedical Metal Materials, College of Materials Science and Engineering, Taiyuan University of Technology, Taiyuan, China; ^3^ Department of Orthopaedic Surgery, Shanghai Jiao Tong University Affiliated Sixth People’s Hospital, Shanghai, China; ^4^ Institute of Translational Medicine, Shanghai University, Shanghai, China; ^5^ Engineering Research Center for Biomedical Materials of Ministry of Education, East China University of Science and Technology, Shanghai, China

**Keywords:** bone marrow mesenchymal stem cells, exosomes, immunomodulation, angiogenesis, osteogenesis

## Abstract

Bone marrow mesenchymal stem cells (BMSCs) have self-renewal, multi-directional differentiation potential, and immune regulation function and are widely used for *de novo* bone formation. However, the wide variation in individual amplification, the potential risk of cancer cell contamination, and the need for culture time significantly limit their widespread use clinically. Alternatively, numerous studies have shown that exosomes secreted by BMSCs in the nanoscale can also affect the functionality of endothelial cells (angiogenesis), macrophages (immunomodulation), and osteoblasts/osteoclasts (osteogenesis), which is a highly promising therapy for osseointegration with pronounced advantages (e.g., safety, high efficiency, and no ethical restrictions). The review aims to summarize the multifaceted effect of BMSCs-derived exosomes on osseointegration and provide reference and basis for rapid and qualified osseointegration.

## 1 Introduction

Osseointegration, a direct structural and functional connection between ordered, living bone and the surface of a load-carrying implant, is critical for implant stability and is considered a prerequisite for implant loading and long-term clinical success of endosseous dental implants. The implant-tissue interface is a highly dynamic region of interaction. This complex interaction involves not only biomaterial and biocompatibility issues but also alteration of the mechanical environment. Multiple cells such as platelets, immune cells, endothelial cells, bone marrow mesenchymal stem cells (BMSCs), osteoblasts, osteoclasts, and osteocytes participate in the process. BMSCs are widely investigated in osseointegration among those cells due to their special characteristics: self-renewability and the ability to differentiate into various cells.

BMSCs are originally found in the bone marrow. These cells proliferate *in vitro* as plastic-adherent heterogeneous cells and have a fibroblast-like morphology. They have the potential for self-replication and multi-differentiation. They can differentiate into cells such as osteoblasts, osteoclasts, and hemangioblasts and have the characteristics of weak autoimmunity and convenient operation (Charbord, 2010). Of note, BMSCs affect the function of immune cells, suppress inflammation, and modulate the injury site microenvironment. It is documented that BMSCs produce the IL-1β receptor, which inhibits B cell differentiation by trapping IL-1, then program macrophage to type 2 (M2) phenotype, which is pro-healing for bone regeneration through releasement of TGF-β, VEGF, and BMP-2. Also, they secrete IL-10 and stimulate the expression of MHC class II, CD45, and CD11 on monocytes; meanwhile, they suppress T cells’ response, increase their differentiation into Treg, and inhibit DC migration and maturation. Moreover, BMSCs inhibit NK cells’ response by secreting factors such as prostaglandin E2, TGF-B1, NO, and IL-6.

Despite BMSCs being a promising candidate as a treatment for osseointegration, apparent problems such as tumorigenesis, fibrosis, pro-inflammatory conditions in the treated bone tissue, cell rejection and injection toxicity, functional erosion, and the restricted ability of BMSCs to differentiate are the barriers that can greatly overwhelm their therapeutic efficacy. Accordingly, researchers endeavor to find and use all the potential of BMSCs to create safe and alternative treatments. Therapeutic amplification of circulatory BMSCs, genomic manipulation of BMSCs, combination therapy of BMSCs with drugs, and use of their exosomes are examples of these efforts. Exosomes are small membranous vesicles (30–150 nm) containing complex RNA and proteins. Currently, they are referred to as discoid vesicles (40–100 nm in diameter). Recent studies have revealed that BMSCs can secrete exosomes to affect osseointegration by affecting the functionality of endothelial cells, macrophages, and osteoblasts. This review aims to summarize the application of exosomes derived from BMSCs in osseointegration and to provide a reference and basis for exosomes in the treatment of osseointegration.

### 1.1 Exosome

#### 1.1.1 Source of exosomes

Exosomes are nanovesicles which having a lipid membrane structure that is bilayer. Exosomes were first discovered in hematopoietic cells such as B cells, dendritic cells, mast cells, T cells, and platelets, but non-hematopoietic cells like epithelial cell types, neurons, and several tumor cells have since been discovered to create them (Cocucci et al., 2009). The exosomes, with a diameter of about 30–150 nm, contain complex bioactive molecules such as RNA and protein, which participate in information exchange and substance exchange between cells (Kalluri, 2016; Pegtel and Gould, 2019).

There are numerous methods available now to collect exosomes. The most popular ones are chromatography, immunomagnetic bead extraction, density gradient centrifugation, and standard ultracentrifugation (Chen et al., 2019). However, these procedures are not commonly employed due to their high equipment dependence, reagent costs, and low yield. Microfluidic devices, NPES, extracellular separation, and membrane-mediated exosome separation technology have developed quickly in recent years. They possess numerous advantages over conventional extraction techniques, including low cost, high efficiency, and ease of use (Yu et al., 2018; Wu X. et al., 2019; Jiang et al., 2020).

#### 1.1.2 Composition of exosomes

**FIGURE 1:**
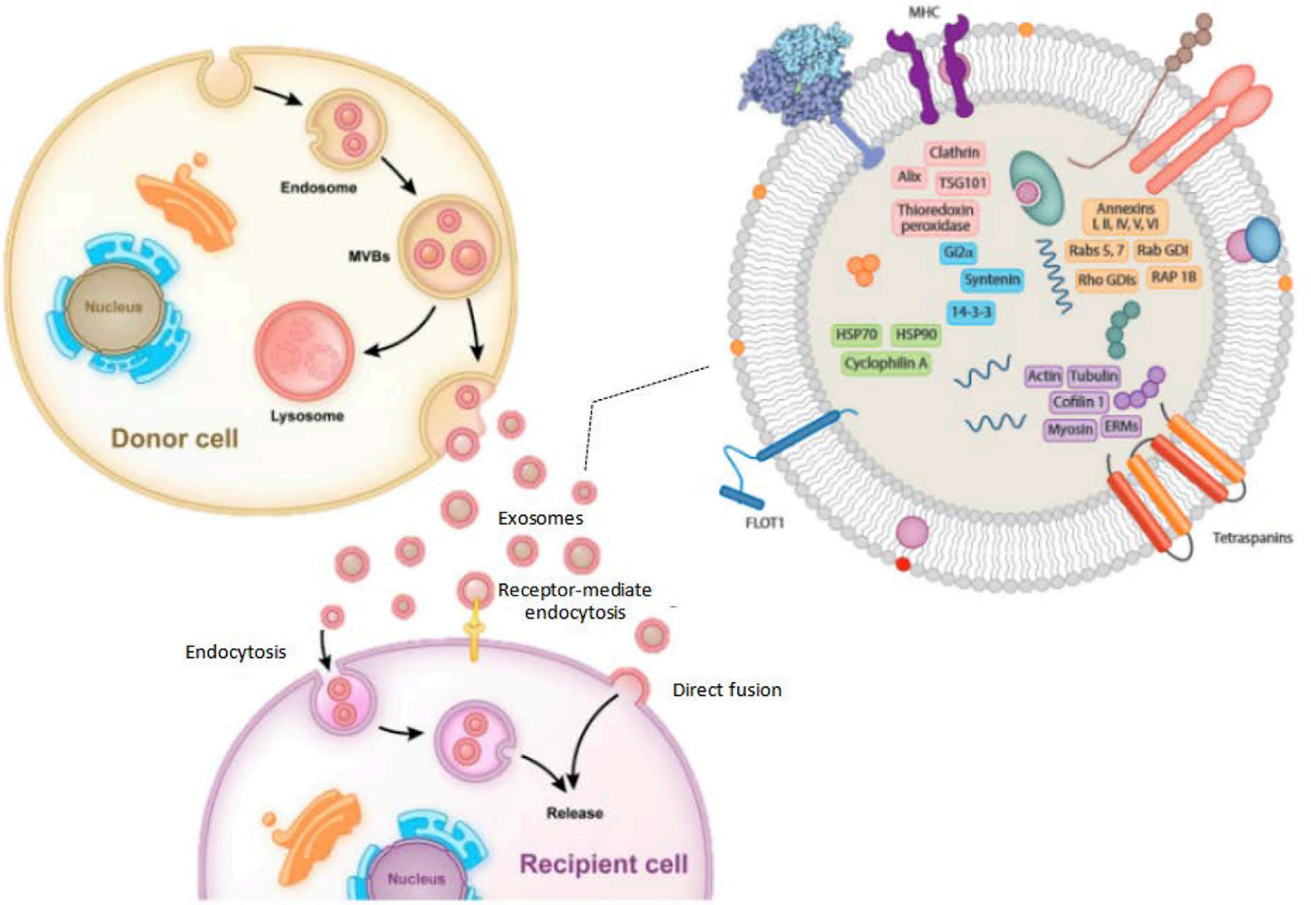
The typical processes of exosome production, secretion, and transfer from donor cells to recipient cells, as well as the structure of exosomes.

The composition of exosomes varies according to the cell type and is rough, as shown in Figure 1. However, exosomes contain many common protein components. The component of exosomes is abundant in cholesterol (particularly B lymphocytes), ceramide (involved in the differentiation of exosomes from lysosomes), various sphingolipids, and phosphoglycerides with long and saturated fatty-acyl chains, in addition to proteins (Subra et al., 2007; Trajkovic et al., 2008). Exocrine bodies are also rich in miRNAs, other non-coding RNAs, and mRNAs. The outer surface of the exosomes also contains glycosyl (Batista et al., 2011). The exosomes usually exclude the endoplasmic reticulum, mitochondria, or nucleoproteins (Schorey and Bhatnagar, 2008).

#### 1.1.3 Function of exosomes

The function of exosomes is determined by the kind of cells they arise, as well as the content of the exosomes’ lipids, carbohydrates, and proteins. Exosomes have some surface components that allow them to attach to distinct cell receptors simultaneously. It is thought that the bioactivity of receptor cells can be regulated by the transport of lipids, proteins, and nucleic acids in the extracellular fluid cycle (Zhang et al., 2015). They also help with antigen presentation through immune cells, which can show anti-inflammatory or pro-inflammatory properties depending on the regulation of antigen-presenting cells. Viruses hijack the exosomal mechanisms of host cells to get beyond the host’s defenses and assist viruses in cross-infection (Schorey and Bhatnagar, 2008). Through the transport of proteins, mRNAs, miRNAs, tumor-derived exosomes may aid in the formation of a systemic cancer-causing microenvironment, encouraging angiogenesis, cell proliferation, and cell survival. Exosome-specific membrane proteins serve as markers for exosome identification and selection, whereas cell type and proteins are unique to each cell type.

Exosomes exist as a cell-free system and have some advantages that cell therapy does not have, such as smaller size, more unitary, long circulatory half-life, low immunogenicity, easy to coat therapeutic substances, easy to cross the blood-brain barrier, easy production and storage, and no tumorigenicity, making it an auspicious choice for regenerative medicine and biomedical treatment (Zhang et al., 2019; Lu et al., 2020).

### 1.2 Four stages of the osseointegration

**FIGURE 2:**
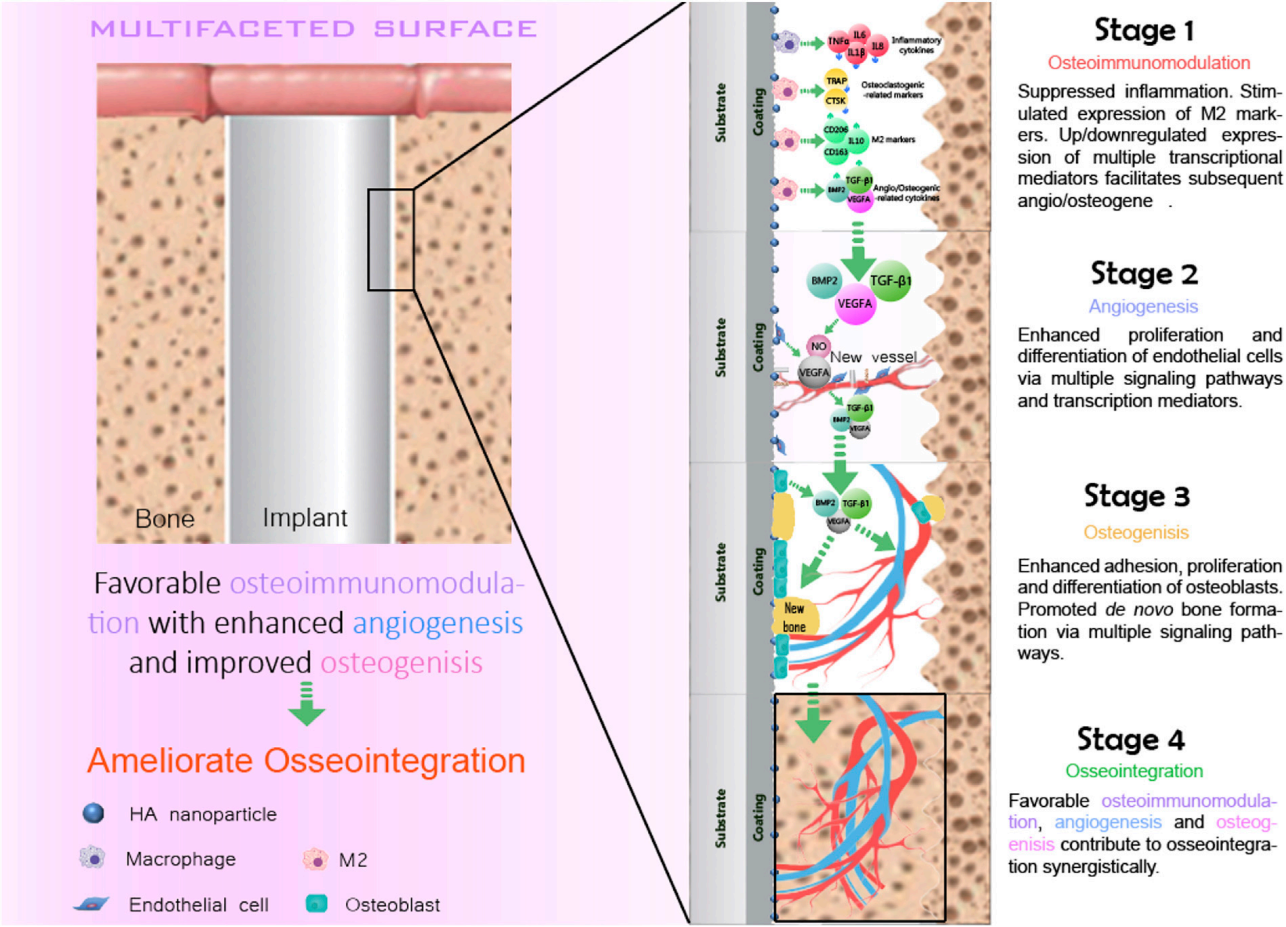
Four stages of the osseointegration.

The concept of osseointegration is the theoretical and biological basis of the function of implants, which refers to the tight connection between the implant material and the bone tissue surrounding it (Albrektsson et al., 2017). Osseointegration is the direct contact between the implant and the surrounding bone tissue without any fibrous connective tissue under an optical microscope (Albrektsson and Ann, 2019; Guglielmotti et al., 2019). It is also called bone fusion. Osseointegration is the key to the stability of implants (Parithimarkalaignan and Padmanabhan, 2013). Osseointegration mainly goes through four overlapping and synergistic phases (Figure 2): the formation of blood clots, immune response, angiogenesis, and the formation of new bones.

#### 1.2.1 The formation of blood clots

The formation of blood clots is the initial stage of osseointegration, which begins within 12–14 h of injury to the bone tissue. During bone implantation, biomaterials inevitably come into direct contact with blood, causing blood clots to form on the surface of the bone before it can regenerate (Shiu et al., 2014). The formation of blood clots is generally divided into two stages: the first stage of endothelial injury activated platelets, platelets in the injury site caused by the aggregation of clumps of structure, and the formation of platelet thrombus, to ensure that no more blood flow (Muthard and Diamond, 2012). But the platelet suppository is relatively loose, firm enough. Then the second stage of formation is initiated, and the clotting cascade begins, causing the prothrombin to turn into thrombin, which acts on the soluble fibrinogen, turning it into insoluble fibrin, which is then cross-linked into a grid structure, a network of platelets, red blood cells, and other components in the blood forms a blood clot. Blood clots provide a matrix for the migration of inflammatory cells, endothelial cells, and fibroblasts. Our recent study also revealed that the blood clot is capable of modulation the osteoimmunomodulation during osseointegration.

#### 1.2.2 Immune response of macrophages

Macrophages are multi-differentiated cells distributed in almost all tissues. It has the functions of phagocytosis, antigen presentation, and secretion of many cytokines (Im et al., 2011). Macrophages play a pivotal role in manipulating the innate immune system, such as regulating inflammation, promoting tissue repair, and maintaining body balance and metabolism. Macrophages have different activation pathways and functional states, among which the inactive macrophages are called the M0 type. Macrophages are highly plastic when activated, and M1 and M2 are two typical phenotypes (Mantovani et al., 2005). Resting macrophages (M0) can be induced to polarize into M1 type macrophages under stimulation of INF-γ and LPS with IL-1, IL-6, TNF-α, and some other pro-inflammatory cytokines are released in significant amounts, and induced nitric oxide synthase, the secretion of these cells confers M1 macrophages with a bactericidal function, which can phagocytize and destroy foreign pathogens, activate adaptive immunity of T cells, and promote inflammatory diseases. Macrophages were induced to M2a by IL-4 or IL-13, M2b by immune complex, and M2c by anti-inflammatory cytokine IL-10. M2 macrophages can release significant quantities of cytokines, including endothelium growth factor, arginase 1, platelet-derived growth factor, transforming growth factor-beta (TGF-β), and IL-10 to relieve inflammation and prevent excessive injury, further facilitate tissue regeneration (Gosselin and Glass, 2014).

#### 1.2.3 Angiogenesis

The process of bone formation in an implant is usually accompanied by angiogenesis, which is the development of new blood vessels from existing capillaries or capillary veins. These include the degradation of the basement membrane during activation, the proliferation and migration of endothelium, and the reconstruction of new blood vessels and vascular networks, a complex process involving multiple molecules from a variety of cells (Parithimarkalaignan and Padmanabhan, 2013; Lugano et al., 2020; Klagsbrun and D’amore, 1991). Angiogenesis is a complex process in which both pro-angiogenic and anti-angiogenic factors coordinate. Under normal circumstances, angiogenesis is in a state of equilibrium. Once this equilibrium is disturbed, the vascular system is activated to cause degeneration of blood vessels by overgrowth or suppression of the vascular system (Folkman, 1997). Angiogenesis is a parallel process with bone formation. Angiogenesis provides the necessary nutrients and oxygen for bone formation. It aids in the development of bones and encourages the growth and differentiation of related cells, although it seldom causes osteogenesis.

#### 1.2.4 New bone formation

After the formation of blood clots, immune response, and angiogenesis, osseointegration is finally accomplished by osteoblasts depositing new bone on the implant’s surface. Osteoblasts are the primary functional cells involved in producing new bones, which persist throughout life but are most active during embryonic bone production and growth (Rutkovskiy et al., 2016). By expressing BMP-2 and TGF-β, osteoblasts boost extracellular matrix production, secretion, and mineralization (Matsunobu et al., 2009). In the balance of osteoblast, osteoclast, and osteoclast, osteoclast can complete the formation of osteoid and the maturation of new bone tissue.

### 1.3 Roles of exosomes derived from BMSCs on osseointegration

A variety of cells, including immune cells, endothelial cells, and BMSCs, play a pivotal role in osseointegration. Exosomes derived from immune cells can express MHC-like molecules and costimulatory molecules on their surface, which regulate a wide range of receptor cells, including epithelial cells, osteoblasts, and osteoclasts. They also promote T lymphocyte activation and participate in and regulate T cell immune responses, affecting osseointegration (Wu R. et al., 2019; Xiong et al., 2021). Exosomes generated from endothelial cells have excellent bone-targeting abilities and can drastically reduce osteoclast development and activity (Li et al., 2016; Zhang et al., 2016). Among them, exosomes derived from BMSCs have received the most research attention due to their close relationship with *de novo* bone formation.

#### 1.3.1 Exosomes on macrophages

The role of immunomodulatory processes in tissue healing, repair, and regeneration has been demonstrated over the past decade. Immune cells, such as T/B cells, macrophages, and neutrophils, play a central role in immune regulation that induces favorable tissue regeneration processes. Among them, macrophages have been evidenced to regulate bone-related cell functionality robustly. Accordingly, this review explicitly addresses the effect of exosomes on the functionality of macrophages in osseointegration (Zheng et al., 2021).

Macrophages, also known as histiocytes, are formed when monocytes in the blood pass through blood vessels. Macrophages are found in practically every organ and are dispersed throughout the body to maintain equilibrium. The form of macrophages is determined by their function (Gordon, 2003). Their processes are usually short and circular or oval. Long pseudopodia with an uneven form are common among active macrophages. Macrophages can divide into M1 and M2 types after activation, which is involved in the inflammatory response. Bone marrow stem cells are beneficial in several investigations. *In vitro* and *in vivo*, BMSCs can change macrophage phenotype from M1 to M2, alter innate and adaptive immune responses, suppress excessive inflammation, and promote tissue repair. Exosomes from BMSCs can cause macrophages to polarize, and exosomes from BMSCs can diminish M2 phenotype of macrophage (Luo et al., 2021). The exosomes of BMSCs not only inhibit the polarization of M1 macrophages but also interfere with their function in secreting pro-inflammatory factors, which can promote the transformation of M1 to M2 and reduce the expression of pro-inflammatory cytokines: TNF-α、IL-1β、IL-6 and IL-8 while increasing the expression of anti-inflammatory cytokine: IL-10.

Under hypoxic conditions, BMSCs secrete a large number of exosomes, which contain numerous functional RNA, the most common of which are miRNAs, such as miR-223, miR-146b, miR126, and miR-199a proteins and microRNAs, these proteins are involved in modulating the posttranscriptional polarization of M1 and M2 macrophages by targeting various transcription factors and participating in the different stages of M2 macrophage differentiation and healing (Arabpour et al., 2021). As a novel macrophage regulator, miR-233 has been shown to play an essential role in the polarization of macrophages and to be a vital regulator of the inflammatory response (Gou et al., 2018). The overexpression of miR-233 can inhibit the polarization of M1, promote the polarization of M2, and increase the proportion of M2 macrophages *in vivo* (Zhuang et al., 2012). We hypothesize that miR-233 may partly play a negative role in the inflammatory pathway by targeting specific signaling pathways, thus downregulating the expression of its downstream pro-inflammatory mediators. PKNOX1 has been experimentally demonstrated to be one of the target genes of miR-233 (Ye et al., 2018). miR-233 directly targets phnox1, inhibits the expression of inflammatory mediators, reduces the levels of IL-1β and IL-18, reduces the infiltration of synovial macrophages, and promotes the polarization of macrophages toward M2 type (Ye, et al., 2018; Xue et al., 2020).

In addition, many miRNAs released from BMSCs have been found to play an essential role in the polarization of macrophages, such as miR-146, miR-155, and miR-222–3p. The miR-146 family includes miR-146a and miR-146b, which play an inflammatory role in many diseases. IL-10 is a potent anti-inflammatory cytokine that induces miR-146b to polarize M1 macrophages by targeting IRF5, whereas miR-146a inhibits IRF5 and reduces inducible nitric oxide synthase, it causes the macrophages to polarize toward M2 (Arabpour, et al., 2021). miR-155 is also a multifunctional miRNA. miR-155 directly affects IL-13Ra1, leading to a decrease in STAT6 activation. Overexpression of miR-155 reduces the phosphorylation of STAT6, resulting in the transformation of M2 to M1, and the expression level of M1 was significantly increased. Compared with M2 macrophages, miR-181a, miR-155, miR-204, and miR-451 were up-regulated, and miR-146a, miR-143, and miR-145 were down-regulated in M1 macrophages.

#### 1.3.2 Exosomes on endothelial cells

Endothelial cells comprise a single layer of endothelial cells that make up the inner wall of blood arteries. They come from the visceral mesoderm. It not only acts as a coagulation barrier between the blood vessel wall and the blood but also ensures that chemicals and cells may readily access the matrix and underlying cells via the blood medium (Lüscher and Barton, 1997). Specific transport systems carry required circulating blood macromolecules from endothelial cells to the subcutaneous regions to fulfill the metabolic demands of surrounding tissue cells.

It is reported that BMSC-derived exosomes may independently activate the VEGF and Hippo signaling pathways (Wang et al., 2021). Hippo-YAP/TAZ signal plays a vital role in angiogenesis by regulating cell-to-cell contact and actin cytoskeleton dynamics. We can modulate the kinetics of the actin cytoskeleton through the time and space differences of LATS1/2, AMOTs, and YAP/TAZ, thus affecting angiogenesis. First, LATS1/2 inactivation inhibits YAP/TAZ phosphorylation, increases its content in the cell nucleus, enhances its transcription capacity, and disrupts and induces changes in EC behavior. Second, AMOTL1 plays a role in localization. It pinpoints the front end of the cell and binds to F-actin, which promotes endothelial cell migration. The activation of Hippo signaling does not depend entirely on VEGF signaling, suggesting that exosomes have a broad and active role in inducing angiogenesis. The results showed that the exosomes enhanced angiogenesis near the tendon-bone junction *in vivo*. In addition, BMSC-exosomes may activate VEGF and Hippo signal pathways independently, and there is no dependent relationship between them.

It has been found that drugs or chemicals can stimulate BMSCs to produce exosomes, such as low doses of DMOG (dimethoxy benzoyl glycine) trigger enhanced angiogenesis activity in the exosomes. DMOG stimulates the release of BMSCs exosomes that promote angiogenesis but not endothelial cell proliferation *in vitro*. DMOG-MSC-exosomes activated AKT/mTOR pathway, stimulated HUVECS angiogenesis, and significantly enhanced p-AKT, mTOR, and p-mTOR expression in HUVECS, thus promoting bone regeneration and angiogenesis.

#### 1.3.3 Exosomes on BMSCs

In addition to hematopoietic stem cells, bone marrow also contains mesenchymal stem cells. BMSCs, also known as pluripotent stromal cells, is an essential member of the stem cell family, a class of pluripotent stem cells that belong to the mesoderm. In addition to being found primarily in bone marrow, BMSCs are also present in connective tissue, such as the placenta, umbilical cord, and fat, as well as in the stroma of organs. It can differentiate into tissue cells such as fat, bone, and cartilage under suitable conditions. Because of its ability for multi-directional differentiation, immune regulation, and self-repair has become the focus of attention in recent years. Under certain induction circumstances *in vivo* or *in vitro*, BMSCs can develop into tissue cells such as fat, bone, cartilage, muscle, tendon, ligament, neuron, liver, heart muscle, and endothelium. Due to aging and disease alterations, the cells might be employed as suitable seed cells for tissue and organ repair. Under specific circumstances, BMSCs can also develop into cardiomyocytes, which can be seen under a microscope as a cell line pulsing spontaneously; BMSCs can be used to differentiate into dermal tissue, which can cover a burn wound. The mesenchymal stem cell has been demonstrated by secreting exosomes to regulate and encourage bone marrow stem cells to develop into bone cells.

The five most abundant microRNAs in BMSC exosomes account for roughly half of the overall microRNAs readout. Many microRNAs have been demonstrated to have physiological effects in previous studies. The pre-microRNAs in BMSCs, some of which may be implicated in MSC biology, may not be released with the exosomes but rather move to other vesicles with distinct characteristics and integrate into them (Chen et al., 2021). miR-143 aids MSC’s immunomodulatory action. MSC differentiation is regulated by the microRNAs: miR-10a and miR-22. miR-10b enhances the migration of BMSCs (Gao et al., 2020). By modulating and fine-tuning proliferation, differentiation, and homing, these microRNAs released by BMSCs may play a role in preserving the stem cell niche. Furthermore, a number of microRNAs found in high abundance in adult exocrine MSC influence cell cycle progression and proliferation (miR-191, miR-222, miR-21, let-7a), angiogenesis (miR-222, miR-21, let-7f), and endothelial cell differentiation (miR-222, miR-21, let-7f, miR-6087). The uptake of these microRNAs at the site of damage may facilitate cell proliferation and induce the development of new blood vessels, both of which are essential for tissue regeneration (Ding et al., 2020; Gaharwar et al., 2020).

Recent research has also looked into the impact of microRNAs on the secretory effect of BMSCs. These microRNAs are found abundant in exosomes and conduct regulatory activities in target cells in addition to regulating the production of proteins released by BMSCs (Chen et al., 2012). The exosomes then support hematopoietic stem cells in the bone marrow, stimulate angiogenesis and blood vessel stabilization, and control the immune system via paracrine functionality (Chung et al., 2010).

#### 1.3.4 Exosomes on osteoblasts and osteoclasts

To maintain the bone mass and calcium homeostasis of the organism, the bone tissue needs to be destroyed and rebuilt continuously. The process of bone remodeling consists of bone resorption and new bone formation. Osteoblasts and osteoclasts are the special cells responsible for bone formation and resorption (Anselme, 2000; Matsuo et al., 2008). The balance between osteoclasts and osteoblasts is the key to maintaining normal bone mass.

Exosomes generated from BMSCs travel via miRNAs are linked to new bone production and osteoblast differentiation (Zhang et al., 2021). miR-935 is one of the most significantly up-regulated miRNAs in human osteoblasts. miR-935 expression is increased in exosomes from bone marrow mesenchymal stem cells, and BMSC-exosomes decrease STAT1 levels by transferring miR-935 to osteoblasts (Yang et al., 2016). Furthermore, inhibiting STAT1 can boost osteoblast proliferation, differentiation, and activity in mineralized nodules, improving osteoblast proliferation and differentiation. miR-935 negatively affects STAT1 and promotes osteogenic differentiation in general (Li et al., 2015).

miR-31a-5p suppresses osteoclast differentiation and increases osteoclast production and resorption while inhibiting bone growth and osteogenesis. miR-31a-5p levels increase considerably when BMSCs age and miR-31a-5p enters the extracellular milieu via exosomes, lowering osteoblast formation and boosting the development of elderly osteoclasts to osteoporosis and other disorders (Fehrer and Lepperdinger, 2005; Zhou et al., 2016). Firstly, miR-31a-5p can mediate BMSC osteogenic and cellular aging via SATB2 and E2F2. miR-31a-5p binds to the 3′UTR of E2F2 to produce SAHF, which promotes cell senescence by boosting the synthesis of SAHF and compromising the function of bone marrow stromal cells, resulting in osteoblast decrease. miR-31a-5p also regulates BMSC osteogenic differentiation via the RhoA pathway.

A recent study further demonstrated that miR-206 from BMSCs exosomes promotes osteoblast proliferation and differentiation in osteoarthritis by inhibiting Elf3 (Huang et al., 2021). miR-199b-5p is produced when BMSCs differentiate into osteoblasts. miR-199b-5p works as an activator in osteoblast differentiation, as miR-199b-5p suppression inhibits differentiation while overexpression promotes it. ALP, Runx2, and ALP activity are all associated with miR-199b-5p. Furthermore, during osteogenesis, the GSK-3/-catenin signaling pathway regulates miR-199b-5p, which affects BMSC osteoblast development. miR-199b-5p acts as a positive regulator in differentiating BMSCs into osteoblasts as a whole.

#### 1.3.5 Drawbacks of exosomes

Exosomes have emerged as appealing candidate therapeutic agents and delivery nanoplatforms due to their unique features and fulfilled biological properties. However, drawbacks such as considerable complexity, low isolation yield, and potential safety concerns significantly hamper their clinical applicability (Ni et al., 2020; Wei et al., 2021).

### 1.4 Engineered biomaterials with BMSCs exosomes

The current gold standard of clinical treatment for osseointegration is autogenous and allogeneic transplantation, although various limiting issues, such as restricted availability, donor site problems, and disease transmission risk, significantly limit their widespread usage. Bone tissue engineering, which uses biomaterials with sophisticated biophysical or biochemical features to circumvent these constraints, has attracted much interest in recent years (Hosseinpour et al., 2017). Ulteriorly, biomaterial engineered with BMSCs exosomes is a highly promising strategy to accelerate osseointegration.

#### 1.4.1 Biophysical combination with BMSCs exosomes

The hydrogel scaffold with high hemostasis and biocompatibility has been regarded as a fulfilled biomaterial. Further combination of BMSCs exosomes with hydrogel scaffold can improve its bioactivity, which can help human umbilical vein endothelial cells grow and multiply more effectively. As one of the best candidate materials for bone repair, mesoporous bioactive glass (MBG) has favorable biological effects such as osteogenesis, angiogenesis, and antimicrobial activity. Previous studies have developed a multilayer MBG scaffold with macro/micro/mesoscopic pores. Micron-scale porous (0.5–2 micron) graded MBG scaffolds can offer BMSCs exosomes with shelter and a large specific surface area. The exosomes loaded MBG showed a satisfying bone regeneration capacity (Liu et al., 2021).

Polyetheretherketone (PEEK) has been employed as an alternative metal implant in bone engineering because of its solid mechanical characteristics, transmissivity, and chemical resistance. It has been reported that BMSCs exosomes can be reversibly anchored to a three-dimensional (3D) porous PEEK surface (Singh et al., 2019). A 3D porous structure was created on the PEEK surface after etching with strong sulfuric acid, which is helpful for exosome loading and distribution and promotes osseointegration. Tannic acid (TA) has many polyphenol groups that can form reversible hydrogen connections between exosomes and PEEK, guaranteeing that exosomes have a long-term release effect (Liu and Xiong, 2021). Exosomes include miRNAs associated with inflammatory regulation, inhibiting the NF-κB pathway and increasing macrophage M2 polarization (Wei et al., 2019). At the same time, Ta-PEEK coated exosomes could create a favorable immune microenvironment for bone regeneration.

#### 1.4.2 Biochemical combination with BMSCs exosomes

HIF-1α can increase BMSCs differentiation into osteoblasts. HIF-1α and RUNX2 interact *via* RUNT domains to enhance angiogenesis and RUNX2 expression by up-regulating VEGF production. RUNX2 is a transcription factor required for osteoblast differentiation and plays a crucial role in the creation and development of bones. RUNX2 is a Wnt/β-catenin signaling pathway target gene that promotes new bone growth by activating the Wnt/β-catenin pathway. In addition, it was found that HIF-1α significantly up-regulates the expression of CXCL12 in the ischemic site, promotes the diffusion of CXCL12 to the periphery, and forms a concentration gradient. Finally, CXCL12/CXCR4 biological axis promotes the mobilization and homing of endothelial progenitor cells to enhance angiogenesis in the ischemic site (Lin et al., 2011; Li et al., 2017). A recent study revealed that BMSC exosomes-HIF-1 was more efficient than BMSC exosomes in promoting BMSC proliferation and osteogenic differentiation. In rats with critical size bone defects, BMSC exosomes-HIF-1 paired with β-TCP scaffold could considerably accelerate the production of new bone and neovascularization differentiation, outperforming BMSC exosomes and β-TCP scaffold (Ying et al., 2020).

Biomaterials with precise chemical ion rep lacements have recently been proposed as a viable method for vascularized bone regeneration (Hu et al., 2012; Li et al., 2014). It has been extensively documented that chemical signals from biomaterials can boost the pro-angiogenic potential of endothelial cells and stimulate osteogenic differentiation of BMSCs (Kholia et al., 2016). More crucially, earlier research has shown that biomaterials’ chemical signals can alter cell-cell contact between nearby cells, resulting in considerably faster tissue regeneration. It is documented that lithium (Li)-BGC promoted human umbilical vein endothelial cells angiogenesis and induced expression of miR-130a in BMSCs-derived exosomes, resulting in PTEN protein down-regulation and Akt pathway activation, resulting in endothelial cell proliferation, migration, and tube formation, as well as up-regulation of angiogenic genes.

Previous research has demonstrated that pretreatment of BMSCs with biochemical stimuli can boost the biological activity of exosomes produced from BMSCs, which is an efficient strategy to improve tissue engineering and regenerative medicine repair efficiency (Wu et al., 2021). Exosomes from IL-1β pretreatment MSCs, for example, induced more IL-10 and TGF-β than those from untreated MSCs (Guo and Wu, 2021; Li et al., 2021). According to another study, exosomes produced from dimethoxy glycine-pretreated BMSCs improved bone repair via boosting angiogenesis (Liang et al., 2019). AKT has been proven essential in angiogenesis by promoting endothelial repair and regeneration. Atorvastatin (ATV) is one of the world’s most widely used prescription cholesterol-lowering drugs (Crismaru et al., 2020). Recent studies have shown that the ATV pretreated BMSCs enhance endothelial angiogenesis by up-regulating the miR-211–3p activation of the ak akenos signaling pathway. The proliferation, migration, tube formation, and VEGF production of HG-injured endothelial cells may all be improved by ATV-Exosomes of BMSCs.

## 2 Conclusion

Exosomes derived from BMSCs exist as a cell-free system, and they possess unique advantages that BMSCs therapy does not have, such as more unitary, long circulatory half-life, low immunogenicity, easy to coat therapeutic substances, easy to cross the blood-brain barrier, easy production and storage, and no tumorigenicity, making it an auspicious choice for regenerative medicine and biomedical treatment. This review describes the effect of exosomes derived from BMSCs on multiple stages of osseointegration and clarifies their multifunctionality in immunomodulation, and osteogenesis. Further combination with biophysical or biochemical cues of biomaterials can boost the efficiency of the exosomes, which is a promising strategy for rapid and qualified osseointegration.
